# What Do We Have to Lose? Offloading Through Moral Technologies: Moral Struggle and Progress

**DOI:** 10.1007/s11948-019-00099-y

**Published:** 2019-03-21

**Authors:** Lily Eva Frank

**Affiliations:** Department of Philosophy and Ethics, School of Innovation Science, Technical University of Eindhoven, 5612 AZ Eindhoven, The Netherlands

**Keywords:** Enhancement, Moral technology, Moral progress, Moral stress, Nudging, Persuasive technology, Behavior change technology

## Abstract

Moral bioenhancement, nudge-designed environments, and ambient persuasive technologies may help people behave more consistently with their deeply held moral convictions. Alternatively, they may aid people in overcoming cognitive and affective limitations that prevent them from appreciating a situation’s moral dimensions. Or they may simply make it easier for them to make the morally right choice by helping them to overcome sources of weakness of will. This paper makes two assumptions. First, technologies to improve people’s moral capacities are realizable. Second, such technologies will actually help people get morality right and behave more consistently with whatever the ‘real’ right thing to do turns out to be. The paper then considers whether or not humanity loses anything valuable, particularly opportunities for moral progress, when being moral is made much easier by eliminating difficult moral deliberation and internal moral struggle. Ultimately, the worry that moral struggle has value as a catalyst for moral progress is rejected. Moral progress is understood here as the discovery and application of new values or sensitization to new sources of harm.

## Introduction: Imagining a (Morally) Brighter Tomorrow

Being moral is difficult, and people often fail to be moral. It requires internal tussles of will between competing desires and commitments, wrestling with hard questions, and even when one has done one’s best, it can leave one with a sense of dissatisfaction, guilt, or uncertainty. A likely and perhaps even desirable feature of the widespread use of moral technologies will be the elimination or, at least, the reduction of the experience of moral struggle. This paper considers and ultimately rejects one way in which moral struggle could have independent value, namely, its role in moral progress.

In what follows is explained the notion of moral technologies being used, gives several examples of them, and describes a hypothetical world in which their use is widespread—a moral Shangri-La. Further clarification is provided about the idea of moral struggle and connects it with the concepts of moral distress, moral overload, and moral residue.

### What are Moral Technologies?

Imagine two worlds. In world one, a moral Shangri-La, people are aided by advanced moral technologies and are able to consistently behave morally while expending little effort to do so. In James Hilton’s novel *Lost Horizon* (1933), Shangri-La is the mythical land of peace, harmony, and happiness that exists hidden away in the mountains of Tibet. In a moral Shangri-La, for example, smart lighting on crowded streets deescalates potentially violent confrontations and robotic diplomats easily negotiate peace processes, etc. World two is similar to our world, where being moral is difficult and people frequently behave badly.

To create a richer picture of these two worlds, imagine waking up in moral Shangri-La. Your alarm clock uses gentle light and ambient smells to gradually rouse you from your sleep, putting you in a mood of openness to new experiences and giving you an optimistic outlook. Your breakfast is vegan, sustainably sourced, and produced under fair-labor conditions. After undergoing a series of treatments, you have lost your taste for animal products and other foods that have a high carbon footprint. In addition, although foods with a greater environmental impact are available, they are placed on the lowest shelves in the grocery store and the sustainable ones are at eye-level, where you always end up grabbing them. Turning on the television, you are reminded that there has been an earthquake in Indonesia. It is essential to combat the insensitivity, or “psychic numbing,” felt towards large numbers of victims, and the preference people have for single, identifiable ones (Slovic 2010). Therefore, you take your daily dose of Empathico. Empathico is a personalized drug, which improves one’s ability to overcome insensitivity to large numbers of victims and feelings of “compassion fatigue” by allowing you to vividly imagine each of them as individuals (Vastfjall et al. 2014).

The television news itself has redesigned its coverage to focus more on single individuals impacted by the disaster rather than spewing out statistics about the suffering of the many thousands of victims. The news coverage includes personalized information, as does your social media feed, about the contributions that your friends, colleagues, and neighbors have made to the Red Cross earthquake relief fund. As you contribute, your social media profile is populated by images of happy children, kittens, puppies, and encouraging messages about your good and generous character.

Once at the office, in front of your computer, your keyboard includes several biofeedback sensors which can detect when your stress level is rising. As you start to write a gossip-filled email to a coworker to relieve your stress, the system releases a barely perceptible breath of air scented with freshly baked apple pie and sends you a pop-up reminder that you have been sitting at the computer for 2 h and it is time for a yoga break. This is just the morning so far; you can imagine the rest of the day in this utopian moral Shangri-La.

Moral Shangri-La is rich with moral technologies. Although the precise meaning of this term is still being contested, the interpretation herein is very broad. Moral technologies include such diverse interventions as moral bioenhancement, nudge-designed environments (from shopping malls and work-places to entire urban landscapes) and policies, ambient persuasive technologies, and social robots designed for moral coaching or advising.^1^ Ingesting a pharmaceutical that changes one’s brain chemistry or undergoing invasive and potentially risky deep brain stimulation for the purposes of moral improvement raise a specific and complex set of ethical questions. The ethical issues they raise are distinct from those posed by less invasive and risky technological interventions, like a wearable fitness tracker that also automatically reminds one to perform a daily act of kindness. However, they are grouped together here because of their shared purpose, moral improvement, and for the reason that a worry about their interference in moral progress can apply to them all.

These technologies may function in at least three ways. First, they may help people behave more consistently with their own deeply held moral convictions. Second, they may assist people in overcoming cognitive and affective limitations and biases that prevent them from fully appreciating a situation’s moral dimensions. There is some evidence, for example, that a feeling of disgust induced by morally irrelevant environmental factors plays a role in the strength of people’s moral judgments (May 2016). Moral technology may serve to dampen or even eliminate this kind of “performance error” (Ibid). Third, they may simply make it easier for people to make the morally right choice, helping them to overcome ego depletion, for example. Ego depletion is the phenomenon that self-control is a finite resource that can be depleted and then requires time to be restored (Baumeister et al. 1998).^2^ Beyond this specific phenomenon, moral technologies may help us overcome the deleterious effects of stress, fatigue, hunger, and other sources of weakness of will.

Over the past decade, speculation about biomedical means of moral enhancement to increase altruism and trust, decrease racial or in-group bias, and decrease aggression has been wide ranging (Persson and Savulescu 2012, 2013).^3^ Some examples among many include drugs such as serotonin and reuptake inhibitors such as Prozac, which in research studies seem to dispose participants to more equally distribute money to other participants in a dictator game (Tse and Bond 2003). Oxytocin, which promotes trusting behavior in economic cooperation games, is another drug that leaves Persson and Savulescu optimistic about future research into the biological basis of morality and the potential avenues for bioenhancement (Zak et al. 2004; Kosfeld et al. 2005). Genetic interventions have also been entertained by some as a means of moral enhancement (Faust 2008), as has deep brain stimulation and transcranial brain stimulation (Kadosh et al. 2010).

In addition to these biomedical methods and more traditional methods of moral education and training, the growing insights of situationist moral psychology point to ways to structure the environmental context, both physical and social, which can be shaped to minimize temptation to do morally bad things and to maximize the likelihood one will behave well (Doris 1998, 2002; Sarkissian 2010; Alfano 2013). For example, altruistic helping behavior towards a stranger is more likely among people in the presence of the pleasant smells of baked goods (Guéguen 2012). Might the streets someday be flooded with these smells?

Situationist moral psychology is closely connected to a critique of virtue ethics and the existence of character traits in the traditional sense. Some situationists have argued that the best way to cope with this sweeping critique of the existence of character traits is to work hard to keep avoiding situations that encourage bad behavior and to seek out those that encourage good behavior (Doris 2002, p. 146). This can be done on an individual basis, but can also be a feature of “supportive social contexts…[and] institutional and legal structures” (Kleingeld 2015, p. 354).

The related fields of persuasive technology and technologies for behavior change more broadly offer even greater opportunities to engineer the environment for maximum moral behavior. Persuasive technologies are “a class of technologies that are intentionally designed to change a person’s attitude or behavior. Importantly, persuasion implies a voluntary change of behavior or attitude or both” (IJsselsteijn et al. 2006, p. 1). At the Technical University of Eindhoven, a large lighting lab is investigating the ways in which colors and intensity of light decrease aggression on streets where there is a busy nightlife; known as the “de-escalate” project, it uses lighting on roads to alter pedestrian perceptions of safety (Haans and De Kort 2012; De Kort et al. 2014). In London, an experiment is being carried out to see if painting adorable faces of babies on the facades of shops could reduce “antisocial behavior” and vandalism on the sidewalks (Rao 2012). This experiment is based on evidence from functional magnetic resonance imaging that shows that looking at still images of nonrelated human infant faces stimulates “specific brain responses …[that] include biological mechanisms that underlie responsiveness and a caring inclination toward young children” (Caria et al. 2012, pp. 891–892).

Empirical research on technology for behavior change and persuasive technology suggests the possibility of devices that provide instant feedback on one’s behavior. For example, in Honda hybrid cars, a device called the eco-assist provides feedback on the driver’s energy usage based on their driving style. This has been shown to change user behavior towards adopting more fuel economy, which is in line with the user’s values (Spahn 2012). The car displays a growing or fading green plant to remind drivers of how their driving, and thus their fuel consumption, impacts the environment (Ibid). Some features of self-driving cars can be seen as utilizing moral technologies; for example, it is likely that cars will be programmed to follow traffic laws, abide by speed limits and not cut other drivers off in aggressive attempts to get ahead (Nyholm and Smids 2016).

Some, but not all, persuasive technologies take advantage of the phenomenon of the “nudge” (Thaler and Sunstein 1999). Nudges, which are factors that influence behavior without one having a clear, rational justification for what one does or being consciously aware of the effect of those factors, often take advantage of well-known cognitive and decision-making biases that people succumb to; for instance, priming and status-quo biases. Nudges can operate independently from anything obviously construed as a technology, including the placement of physical objects on a store shelf or the arrangement of links at a website, the creation of opt-in or opt-out systems, etc.

A fundamental question in the design and assessment of moral technologies is which values and whose values, norms, conception of the right, etc. should be built into them. The same questions arise in broader discussions of persuasive technologies or nudge enriched environments (Smith and McPhereson 2009). What one person or group sees as a moral improvement to behavior or attitudes, may be seen as a sign of moral decay or decline by another.^4^ The description of moral Shangri-La I presented as well as the description of sample moral technologies appears to assume that there is consensus on the ethical issues, which is not always the case. However, this complication is set aside in this paper, assuming that the moral technologies in this case are those which have latched on to the ‘right’ moral theories, the relevant values, norms, and principles. Whether or not this is a metaethical possibility is beyond the scope of the argument.

### Moral Struggle is Missing in Moral Shangri-La

In the following discussion two assumptions are made. First, technologies to improve individuals’ moral capacities and behaviors are realizable. Second, such technologies will actually help them get morality right and behave more consistently with whatever the ‘real’ right thing to do turns out to be. In a sense, these technologies will allow people to engage in a kind of moral offloading. Just as people can offload certain cognitive tasks, such as keeping track of appointments on the calendar application on their smart phones, the phenomenon of moral offloading is getting machines to do moral work for people in a variety of ways, be it care work, tactful communication, or automatically sending birthday cards (for an example of this phenomenon, see Lee et al. 2017); these technologies will allow them to delegate certain internal moral processes to the environment, a technology, or other kind of intervention. Given these assumptions, the following considers whether or not humanity loses anything valuable when technology makes being moral and behaving morally much easier, eliminating difficult moral deliberation and internal moral struggle.

One way in which moral struggle could have independent value, in addition to the value it has insofar as it helps people to know what the right thing to do is or to do the right thing, is as a catalyst for moral progress. Moral progress, itself a highly contested topic, is understood here as the discovery and application of new values or sensitization to new sources of harm. This highlights the question: is moral progress possible in a moral Shangri-La, without moral struggle?

Part of what makes moral technologies promising is the prospect of eliminating or reducing moral struggle. Moral struggle can be characterized in an ecumenical way, one that is neutral about moral psychology. It can be characterized by any moral psychological theory, including cognitivist, noncognitivist, hybrid view, or something in between or entirely different. This is because moral struggle is generally accepted as part of what it is that any metaethical or moral psychological theory needs to explain. It is especially important that these theories account for the fact that individuals sometimes, perhaps even often, fail to act in accordance with what they think is the best way to act from a moral perspective. Therefore, whether a theory takes moral judgments to be belief-like mental states or affective, emotion-like mental states, it must still be able to account for weakness of will and the experience of mismatch between one’s moral commitments (however that is construed) and one’s behavior.

Intuitively, a wide range of experiences can be associated with failing to act in ways which, according to one’s own moral commitments and principles, were the right ways to act. For example, an agents may hold the belief that they should refrain from an action, but a conflicting desire overwhelms their desire to act in accordance with their moral conviction and they perform the forbidden action anyway. On this view, moral struggle includes experiences associated with traditional notions of weakness of will but can include cases in which one ruminates extensively over what the right course of action actually is, and in the end, one is not sure whether or not one has done well from a moral point of view. Moral struggle can also include the experience of guilt or regret that one may feel looking back at bad moral decisions or failures to act. Moral struggle of this kind can also arise as a consequence of automatic decisions that upon reflection have moral significance but are lost in the course of everyday life.

Three examples of moral struggle that have been discussed in the literature are moral distress, moral overload, and moral residue. Moral distress, specifically in the context of nursing, is a well-studied example of moral struggle. In that context, moral distress is

[t]he painful feelings & the psychological disequilibrium that occurs when nurses are conscious of the morally appropriate action a situation requires but cannot carry out that action because of institutionalized obstacles…including lack of time, supervisory reluctance, an inhibiting medical power structure, institution policy, or legal constraints…the person feels frustration, anger and anxiety when faced with institutional obstacles and interpersonal conflict about values (Corley et al. 2005, p. 382).

Potential consequences of moral distress include the experience of becoming “numbed” to “ethically challenging situations.” This can occur to the point that nurses “may no longer recognize or engage in clinical situations requiring moral sensitivity” (Epstein and Delgado 2010). It can lead to nurses contemplating leaving their job or the field of nursing altogether (Corley 1995; Hamric and Blackhall 2007). There is also evidence to suggest a positive relationship between moral distress and burnout (Meltzer and Huckabay 2004). This can lead to conscientious objection or whistleblowing with respect to practices nurses find morally unacceptable or problematic (Catlin et al. 2008).

The phenomena of moral overload and moral residue are closely related and both attempt to capture the personal experience of leaving unfulfilled moral obligations that one takes oneself to have or having acted, reluctantly, contrary to what one takes to be morally required (Kuran 1999; Van den Hoven et al. 2012). Moral overload arises in situations in which one’s moral obligations outstrip one’s material resources, either physical or financial (Kuran 1999, p. 233). Because one cannot execute what one takes to be one’s obligations or duties, one is left with a feeling of “moral dissonance, psychological discomfort” (Ibid). For example, Jaap may believe he is obligated to attend a protest resisting an unjust policy in the United States. However, the cost of an airline ticket home is more than Jaap’s current bank account balance. Here, his financial constraints prevent him from fulfilling a duty.

The existence of moral dilemmas gives rise to the phenomenon of moral residue. One account of what a true moral dilemma is, is a situation in which there are several courses of action open to the person, none of which is morally acceptable. Or to put it slightly differently, after having considered one’s prima facie reasons for several courses of action, one finds that one has several competing things they consider morally required courses of action that are incompatible with each other. In these kinds of cases, a person may experience distress because “there remains a duty unfulfilled, a value commitment not met” (Van den Hoven et al. 2012, p. 146; see also Webster and Bayliss 2000).

Moral distress, overload, and residue are negative phenomena from a first-person perspective because they involve discomfort or stress and can have potentially negative consequences on the individual experiencing them. However, in the case of moral distress, it may be an appropriate affective response to a situation in which one witnesses or participates in wrongdoing and feels powerless to change it. In the case of moral overload and residue, these may also be the appropriate responses of agents who take their ethical commitments seriously in a world in which values and duties can genuinely conflict. Research shows that moral distress in nurses can sometimes precipitate taking a bold and potentially risky stand as a whistleblower, or conscientious objector to an immoral practice.

### From Moral Struggle to Moral Progress?

Whether or not moral struggle is a psychologically positive experience to have in terms of individual well-being, what matters for this paper is its relationship to moral progress. Before setting out what that relationship might be like, the following clarifies that the account of moral progress being relied on in this paper comes from the work of Jamieson (2002), who builds on what he calls the naïve view of moral progress, which states that: “[m]oral progress occurs when a subsequent state of affairs is better than a preceding one, or when right acts become increasingly prevalent” (Jamieson 2002, p. 318). Instead of constructing a robust theory of what moral progress is, Jamieson defends an index of moral progress, which includes such things “as involving the abolition of war and slavery, the reduction of poverty and class privilege, the extension of liberty, the empowerment of marginalized groups, and respect for animals and nature” (Ibid).

Jamieson proposes an account of moral progress than can be consistent not only with several different normative theories, or theories of value, but also with several different metaethical theories. One does not have to be a realist to endorse this view of moral progress.

To defend the independent value of moral struggle, one could argue that in a moral Shangri-La (rich with moral technology), moral progress is stifled precisely because of the elimination or reduction of moral struggle. People have fewer opportunities for introspection and for self-doubt and for doubt about the collective ways and customs of their communities. In a moral Shangri-La, people do not have to think about morality very much, nor do they commit to it significant mental resources. This may engender a kind of moral complacency. In the absence of this rumination, people may be less willing to take moral risks or engage in moral experimentation.

To adequately address the issue of the ways in which moral struggle may require moral progress would require an account of when moral progress happens and under what conditions, both for the individual and for societies or groups. Such an investigation is beyond the scope of this paper. For the purposes of this paper four potential types of relationships between moral struggle and moral progress are considered.

The first has to do with prompting reflection. It is possible that moral emotions such as guilt, produced by weakness of will, may lead to a broader reflection on one’s habits, priorities, and values that have moral relevance. For example, guilt at cancelling plans with a friend at the last minute without a good reason might lead one to think about the value of friendship in life more generally or whether or not it is permissible to lie to one’s friend and tell them you are sick, rather than tell them the truth.

Second, moral struggle may prompt action, as in the case of moral distress among nurses. This also seems to be the case in situations involving moral overload. In fact, Joroen van den Hoven and colleagues argue that both of these experiences can spur the creation of new technologies to help overcome the physical or financial limitations that prevent people from fulfilling their duties. Or, in the case of moral residue, the unpleasant experience which stems from the existence of a moral dilemma may prompt one to find ways to dissolve the dilemma altogether, they suggest, potentially through technological innovations.

The third and related type of relationship between moral struggle and moral progress has to do with the diversity of responses to difficult moral problems. The worry here is not that in a moral Shangri-La, filled with moral technology, differing moral perspectives would be actively forbidden or a significant loss of freedom would occur. Instead, the concern is that when people go through experiences of moral struggle, these have the potential to stimulate people to rethink their moral assumptions, the scope of their moral concern, or the kinds of consequences that their actions or inactions may have. Thus, experiencing moral struggle may be a catalyst for new and radical moral innovations; for example, strategies of nonviolent resistance to authoritarian political regimes that have emerged from movements and leaders of movements during times of great inner moral conflict. As individuals offload moral decision-making to technologies or the built environment, the worry might arise that some of the skills, cognitive or affective, necessary for this kind of moral innovation will be lost.

Fourth, perhaps the role of moral struggle in prompting moral progress is in preventing moral complacency. In the absence of the experience of wrestling with competing values and obligations and being epistemically challenged regarding the right course of action to take, individuals may be lulled into the mindset that they (their characters, behavioral patterns, etc.) and the world around them (institutions, financial arrangements, cultural attitudes) are as good as they are going to be and that there is no need or even room, perhaps, for improvement.

### Two arguments Against the Value of Moral Struggle for Moral Progress

Although these sound like plausible attempts at explaining how moral struggle is needed for moral progress and thus has value for this reason, the claim fails for two independent reasons. First, it depends on the assumption that in a moral Shangri-La, there is room for moral progress. Moral progress is only possible if at the present time the world is in a less-than-ideal moral state. The defender of a moral Shangri-La can argue that with technological enhancements individuals and their world will be as good as they can possibly be from a moral perspective or at least that, with the help of moral technologies, people have all of the relevant knowledge and tools for continuing to improve their moral world, in the absence of struggle.

There are some problems with this response. It makes the implausible claim that with the help of moral technologies of various sorts, people will be able to reach a state of moral perfection, which seems overly optimistic. One reason to be pessimistic about this is that what constitutes moral perfection is only available from the perspective of a particular moral theory. On a hedonistic utilitarian account, for example, a world in which no further moral progress is possible might require that the suffering of all sentient beings is at the lowest possible attainable level and their pleasure is maximized. Therefore, while it is a problematic assumption that a moral Shangri-La needs moral progress, it can be overcome.

There is a second response to the claim that moral struggle is needed for moral progress. There are other ways of achieving moral progress, perhaps even more effective ways of doing so than moral struggle. Thus, it is possible to achieve moral progress in a moral Shangri-La. The moral technologies used there, far from interfering with moral progress, may aid moral progress. If it is granted that thinking about morality and being moral is effortful and requires the expenditure of mental resources, moral technology can reduce this effort.

One way it does so is through the automation of many moral decisions. This can reduce an individual’s overall mental burden, thus contributing to making better decisions when they are not automated. This could be especially important for people whose decisions can have serious impacts on the well-being of others: physicians and nurses, soldiers, police, lawmakers, and so on. In medicine, for example, many decisions and behaviors of ethical import are made on a daily basis, requiring varying degrees of attention, concentration, and effort. In a moral Shangri-La, routine matters of ethical significance are made easier through technology, say, an electronic “nudge” that reminds the physician to warmly greet her patient with a handshake when they meet. This may free the physician to dedicate more of their time and energy to assessing the decision-making capacity of a mildly demented patient to refuse life-saving medical care, a decision with arguably greater moral significance.

In the absence of moral struggle, in a moral Shangri-La moral progress can also come in the form of gaining greater ethical insights and heightened sensitivities to previously overlooked moral issues and fine-grained distinctions. There are two mechanisms that might work here. First, being consistently moral in one respect, as a result of using a moral technology, may draw attention to analogous cases in which people previously did not realize they were behaving immorally. They might then realize that they would be inconsistent in continuing to behave as they currently are, given that they accept the underlying justification for the use of their moral technology.

For example, before the widespread acceptance and partial legal enshrinement of the rights of lesbian, gay, and bisexual (LGB) individuals in the United States, the issue of the abuses of the rights of transgendered persons was largely ignored. With the first form of moral progress, recognition and protection of LGB rights, gradually comes a cultural acceptance of transgendered people as well. Being consistently moral in one respect may draw attention to analogous cases in which people previously didn’t realize they were behaving immorally. They might then realize that they would be inconsistent in continuing to behave as they currently are. People may also come to accept more types of persons or beings into the sphere of moral consideration with the increasing of impartiality in their judgments about people as a result of this same process (see Singer 2011).

A second mechanism of moral technology use, and the accompanying reduction in moral struggle that might still spur moral progress, has to with the benefits that moral technologies can provide, such as lower stress levels and greater individual well-being. While these benefits may not seem like straightforwardly “moral gains,” they can be understood in that way for direct and indirect reasons. Before such an argument can be made, however, some suggestive evidence is offered that moral technologies and the reduction of moral struggle could indeed have these kinds of effects on well-being and stress. Wilhelm Hoffmann and colleagues have demonstrated that individuals with higher levels of self-control experience higher degrees of momentary positive affect as well as higher overall reported life satisfaction (Hofmann et al. 2014). The fact that both of these measures of well-being are positively correlated to self-control is telling; it weighs against the picture of the highly self-controlled person as one who forgoes momentary pleasures and thus experiences a reduction in positive affect, and instead achieves long-term goals.

This research also suggests that what mediates this positive relationship is the fact that unhappiness and stress are often produced by the experience of conflicting goals, especially in cases where the individual greatly values one goal and values another less, whether these goals are self-interested (an individual wants to smoke and they want to quit smoking) or more overtly moral in nature (an individual wants to remain comfortably in their bus seat and they want to be the kind of person who gives up a seat for the elderly).

Individuals with greater self-control are less troubled by these conflicts. It is not clear if this is so because these individuals tend to structure their lives in such a way that they are simply exposed to fewer temptations, or because for these individuals it is easier to resist temptation. Both of these capacities, structuring one’s life and environment and the ability to resist temptation, can be extended and improved through the use of moral technologies. Hoffman and colleagues have even gone so far as to call these “virtue/vice” conflicts. Individuals with greater self-control seem to be less troubled by these conflicts, although it is not entirely understood why. It may be that they structure their lives in such a way that they are simply exposed to fewer temptations than others, or it may be easier for these individuals to resist temptation for other reasons (Hofmann et al. 2014). If this analysis is correct, then the use of moral technologies may also usher in lower stress levels and greater individual well-being.

This can be construed as moral progress directly when returning to Jamieson’s broad notion of progress compatible with many different normative theories. Certainly for a hedonist, the reduction in stress and increase in individual well-being could be understood as a moral improvement. The empirical evidence regarding whether or not happy, less-stressed people also tend to behave better morally is extremely inconclusive. Therefore, the possibility that this development would have a positive moral spillover effect remains open. Stress does seem to alter ethical decision-making. There has been a limited amount of research on this topic, with contradictory results (Starcke et al. 2011, 2012; Youssef et al. 2012; Buchanan and Preston 2014).

### An Aristotelian Objection and a Kantian Objection

The arguments canvassed so far about the value of moral struggle for moral progress do not rule out the possibility that something else of value is lost when moral struggle is eliminated through the use of moral technologies. There are, for example, Kantian-style and Aristotelian-style objections, broadly speaking, to consider.

The Aristotelian-style objection has to do with the extent to which the automaticity of being moral interferes with the criteria for behaving virtuously. Not only must one act well, at the right time and in the right situations, but one must also perform the virtuous activity for its own sake (Aristotle NE 6.5 1140b; 6.12 1144a). Virtuous agents also know what they are doing, that they are performing a virtuous act when they perform it.

Both of these criteria may seem hard to fulfill in a moral Shangri-La permeated with moral technology. Whether someone is choosing action for its own sake is obscured by the introduction of a mind-altering drug or an environment dosed with smells that are conducive to kindness. Of course, not all moral technologies will function in this way, and moral technologies can also be imagined that do not function in this way. Second, what it means to do something for its own sake, and how to reconcile this requirement with a eudaimonistic account of the virtuous life, is notoriously difficult (see Hurka 2013; Korsgaard 2008).

Second, moral technologies may make it harder for those doing the virtuous action to know that they are doing the virtuous action. One of the purposes of moral technologies, as they have been imagined here, is to free up one’s mental space from thinking about right and wrong in certain situations and simply be able to go with the flow with less scrutinizing and policing of one’s motives and actions. Thus it appears that the possibility that people will do virtuous acts, without being consciously aware of them as such, will increase. In addition, according to one straightforward interpretation of Aristotle (2014), this would exclude them from acting out of virtuous character.

There is room for a defender of the value of moral technology to push back in a variety of ways. The first objection has to do with choosing the virtuous action for its own sake. What it means to choose the virtuous action for its own sake is a vexing question in Aristotle scholarship, especially because eudemonistic ethics like Aristotle’s seems to emphasize that the virtuous life and the flourishing or happy life are one in the same (Rogers 1994; Ackrill 2010; Luthra 2015). On some readings acquiring virtue is done so that one may live a flourishing life. It is beyond the scope of this paper to discuss these subtleties. However, as one is practicing the virtues and before one has acquired them, one may be performing the virtuous action for other reasons than for their own sake, with the hope that embodying the virtues will eventually become one’s second nature. The details of the design, functioning, and psychological impacts of moral technologies are relevant here for the following reason. The technologies may function as a kind of training aid, helping people acquire virtues through habituation.^5^ In that case they support the development of true virtue.

The second Aristotelian objection, the worry that moral technology interferes with one’s ability to knowingly engage in virtuous action, can in part be remedied through technology design. For example, environments enriched with moral technologies or the devices themselves can periodically remind users of their function and of the moral value of engaging in a particular action or taking a certain attitude. Non-technical education can also play a role in making sure that users understand the underlying reasons for the interventions they are subject to or with which they are engaging. This objection can also be defused from a more theoretical perspective. One does not need to be concurrently aware that one’s action is virtuous; instead, one can hold the belief that actions that conform to certain kinds of standards (or whatever the criteria might be) are virtuous, and be aware that one consensually modifies one’s environment to make these actions likely. However, a fuller explanation of how this can be made consistent with Aristotelian moral psychology is needed.

The Kantian-style worry is that actions performed as a result of interaction with moral technologies may not have moral worth at all because they are not performed out of good will or out of respect for the moral law (Kant 1785/2012, Chapt. 1), and are performed merely in accordance with duty rather than from duty. Basic Kantian ethics states that morality must, therefore, be founded on the exercise of specific human capacities that individuals are responsible for and of which they are in control. If what matters morally is simply having the right kind of disposition—kindly or sympathetic—being moral would be out of some people’s reach because their emotional dispositions are largely out of their control. What all adult humans share is a capacity to reason and act autonomously; thus this must form the foundation of morality. The Kantian worry is that one’s donation to the earthquake victims’ fund will not be made out of respect for the duty one has to benefit others, but out of some animal-like impulse mediated by technology. In this way one becomes like Kant’s shopkeeper, who treats his customer fairly out of prudence or out of love for his fellow humans. His actions do not have moral worth, as they would if they were motivated by duty.

This kind of concern is echoed in John Harris’ criticism of moral enhancement through the direct modulation of emotions or other noncognitive means in ways that he sees as interfering with human freedom (Harris 2013a).^6^ In debate with Tom Douglas, Igmar Person, and Julian Savulescu, Harris has pointed out that many so-called moral enhancements will “not necessarily lead to morally better outcomes” (Harris 2013b, p. 288). This is because of the specific view of moral decisions he espouses.

According to Harris, moral decisions depend on a rational process that issues moral judgments according to evidentiary and justificatory standards and the absence of arbitrary personal preferences or biases (Harris 2013b, p. 289). Thus, the kind of decisions people would make and actions they would take under the influence of certain kinds of moral enhancement would fail to qualify as moral decisions, even if they make the world a happier or less-violent place. Decisions and actions that have “moral consequences,” according to Harris, do not necessarily make them moral decisions.

Full responses to the Kantian and Harris style objection are also beyond this paper’s scope. However, there are potential avenues for responses. Perhaps, as motivational externalists argue, in order for someone to be motivated to act on their moral belief they must possess a belief-like state with moral content and a corresponding affective or desire-like state(s) (Miller 2008). Moral technologies may contribute to the affective or desire-like component of motivation without undermining an individual’s attitude of respect for the moral law or duty. Another possibility is that moral technologies serve, in some cases, to remind one of one’s duties in particular instances or prompt one to act on them. This possibility would also not undermine the moral value of the act on Kantian standards as long as one holds a standing commitment to respect for the moral law and duty. Living with the help of moral technologies can be done out of respect for the moral law itself.

Aristotelian or Kantian objections underlie the importance of moral psychology in the design and deployment of moral technologies. At the least, they suggest that it is necessary get moral psychology right before moving on with the project.

## Conclusion and Future Work

The paper has argued that one of the central features of a world saturated with moral technologies will be a reduction in moral struggle, as it becomes easier and easier to do the right, good, or virtuous thing. Whether or not moral struggle has independent value remains an unanswered question. It is a question that demands to be explored in future work on moral technologies, as does, specifically, the phenomenon of moral offloading. This paper, however, considered whether moral struggle is necessary for moral progress and concluded that it is not. There is also exciting work to be done on expanding on the notions of moral residue and overload, especially with respect to the role of technology. Joroen Van den Hoven, Gert-Jan Lokhorst, and Ibo Van de Poel see the potential for technological progress and moral progress to be mutually reinforcing processes.

## Footnotes

1 See for example Borenstein and Arkin (2016) and Klincewicz (2016).

2 The existence of this phenomenon has been recently criticized. See Carter and McCullough (2014).

3 In addition to moral *bio*enhancement, first Marta Solowska, Michał Klincewicz, and Lily Frank have suggested updates to traditional the forms of moral development and education of children that are empirically informed by the Cognitive-Affective Personality System (Mischel and Shoda 1995; Klincewicz et al. 2018).

4 I thank an anonymous reviewer for suggesting I note this.

5 This point was made particularly clear to me in conversation with Saskia Nagel and Andreas Spahn.

6 Harris (2011) does argue in favor of cognitive enhancement as a form of moral enhancement.
